# Community-acquired pneumonia alters circadian regulation of cardiac electrophysiology in children

**DOI:** 10.1007/s00431-026-07006-1

**Published:** 2026-04-30

**Authors:** Gul Sahika Gokdemir, Adem Aktan, Özhan Orhan, Feyat Tunç, Mehmet Tahir Gokdemir

**Affiliations:** 1 Department of Physiology, Faculty of Medicine, Mardin Artuklu University, Mardin, Turkey; 2 Department of Cardiology, Faculty of Medicine, Mardin Artuklu University, Mardin, Turkey; 3 Department of Pediatrics, Faculty of Medicine, Mardin Artuklu University, Mardin, Turkey; 4 Department of Pediatrics, Batman Training and Research Hospital, Batman, Turkey; 5 Department of Emergency Medicine, Faculty of Medicine, Mardin Artuklu University, Mardin, Turkey

**Keywords:** Community-acquired pneumonia, Child, Electrocardiography, Circadian Rhythm, QT Interval, Ventricular Repolarization

## Abstract

The effects of community-acquired pneumonia (CAP) on cardiac electrophysiology have been described in children; however, their relationship with physiological circadian regulation remains unclear. This study aimed to evaluate morning–evening variations in electrocardiographic parameters in children with CAP compared to healthy controls. Thirty-six children diagnosed with CAP and 47 healthy controls were included. Electrocardiographic recordings were obtained in the morning and evening. Heart rate, atrial conduction parameters, and ventricular repolarization indices were analyzed to assess time effects, group differences, and time × group interactions. Children with CAP exhibited higher heart rates and shorter atrial conduction parameters than controls at both time points. Ventricular repolarization indices were increased in the pneumonia group. Tp-e/QT and Tp-e/QTc ratios were higher in children with CAP but did not show significant temporal variation. The frontal QRS–T angle demonstrated limited diurnal variation in healthy controls, whereas no significant temporal change was observed in the pneumonia group. Associations between Tp-e/QT, Tp-e/QTc, and QTc differed according to measurement time.

*Conclusion*: Children with CAP demonstrate alterations in atrial conduction and ventricular repolarization compared with healthy peers. These findings suggest that CAP may be associated with temporal changes in specific electrophysiological relationships rather than a complete loss of physiological circadian regulation.

**Graphical Abstract:**

This figure illustrates the subclinical cardiac effects and selective circadian modulation of community-acquired pneumonia (CAP) in children. Children with CAP show increased heart rate, decreased atrial conduction (PR interval), and altered Tp-e ratios. Despite preserved circadian regulation, these functional and reversible changes highlight the need for multidimensional, time-sensitive ECG assessment.

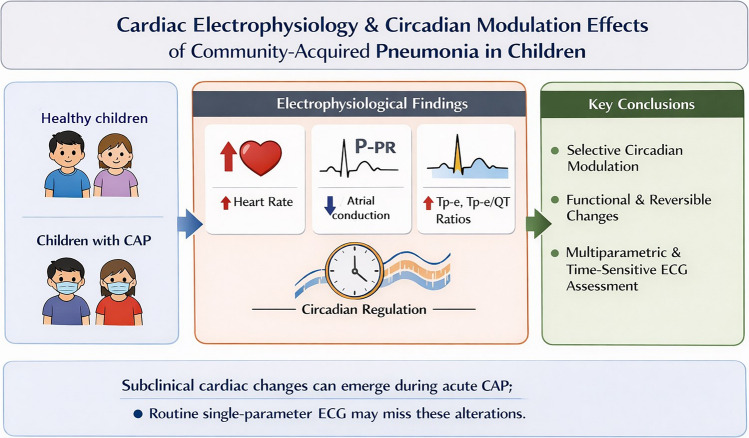

**Table:** 

**What is Known:** • *Community-acquired pneumonia (CAP) can aff ect cardiac electrophysiological parameters in children.* • *Circadian variation infl uences electrocardiographic parameters under physiological conditions.*
**What is New:** • *Children with CAP show alterations in atrial conduction and ventricular repolarization compared with healthycontrols.* • *CAP is associated with changes in temporal electrophysiological relationships rather than a complete disruption ofcircadian patterns.*

## Introduction

Pneumonia is one of the leading causes of morbidity and hospitalization worldwide in childhood, and the disease is not limited to pulmonary involvement but is accompanied by significant systemic inflammatory and autonomic responses [1, 2]. Community-acquired pneumonia (CAP) is defined as pneumonia that develops in previously healthy individuals who have not been hospitalized recently [3]. Increased proinflammatory cytokine release during acute infection, sympathetic nervous system activation, and hypoxemia that may accompany pneumonia can affect cardiac electrophysiological characteristics even in children without structural heart disease [3–5].

Cardiac electrical activity is regulated by the circadian rhythm, and this regulation is primarily dependent on the balance between the sympathetic and parasympathetic nervous systems [6–9]. In acute inflammatory conditions, this physiological balance may be disrupted, leading to an increase in myocardial electrical heterogeneity [7]. In electrocardiography (ECG), P wave duration, PR interval, and QRS duration reflect cardiac conduction, while QTc interval, Tp-e interval, and Tp-e dispersion are considered noninvasive indicators of ventricular repolarization heterogeneity [10–13]. Inflammatory mediators and autonomic dysfunction have been shown to affect these parameters independently of electrolyte disturbances [14–17].

The effect of acute inflammatory diseases in childhood on the circadian regulation of cardiac electrophysiology has not been sufficiently elucidated [18–21]. Therefore, this study aimed to evaluate the effects of pneumonia on the physiological circadian regulation of cardiac conduction and ventricular repolarization by comparing electrocardiographic parameters obtained in the morning and evening in children diagnosed with CAP and healthy controls.

## Materials and methods

### Patients’ medical reports

This prospective, observational, two-center case–control study was conducted in accordance with the principles of the Declaration of Helsinki. Ethical approval was obtained from the Scientific Research Ethics Committee of Batman Training and Research Hospital (approval date: 02/20/2025; approval number: 415). Written informed consent was obtained from the parents or legal guardians of all participants prior to enrollment.

Sixty children diagnosed with CAP were evaluated for the study. After excluding cases with electrolyte imbalance that could affect electrocardiographic measurements, 36 children in the pneumonia group and 47 healthy children matched for age and gender (aged 3–18 years) were included in the study. All patients in the pneumonia group were hospitalized with a diagnosis of CAP. The diagnosis was based on a combined assessment of clinical findings such as fever, cough, tachypnea, respiratory distress, and abnormal lung auscultation, together with chest radiography performed in all patients, demonstrating findings consistent with pneumonia. Etiological classification (viral or bacterial) was based on clinical evaluation, laboratory findings, and radiological assessment. Blood cultures were taken when deemed clinically necessary; however, no patients had positive blood cultures at the time of ECG recording. Both viral and bacterial cases were included in the study. No standard scoring system was used to assess the severity of pneumonia, and patients hospitalized due to hypoxemia but with a clinically stable course were included in the study. To obtain a homogeneous patient population, patients requiring intensive care, meeting the criteria for severe pneumonia, or developing a need for mechanical ventilation were excluded from the study. The study group consisted of CAP cases without severe disease who were monitored at the ward level. Children with congenital heart disease, known arrhythmias, systemic inflammatory or autoimmune diseases, chronic lung diseases, kidney or liver dysfunction, electrolyte imbalance, or use of drugs that could affect cardiac conduction and repolarization were excluded from the study. Children aged 0–2 years were also excluded from the analysis because cardiac repolarization parameters can physiologically vary in early childhood, increasing measurement variability.

Data were obtained from Mardin Training and Research Hospital and Batman Training and Research Hospital. ECG measurements were taken from patients with pneumonia on the first day of hospitalization. For each participant, two standard 12-lead ECG recordings were taken on the same day, one in the morning and one in the evening. No additional ECG recordings were taken. ECG measurements in the control group were taken at the same time points. To assess the potential circadian modulation of cardiac electrophysiology, these time points were chosen to reflect the daily variability of autonomic nervous system activity throughout the day. The literature reports that ECG parameters such as heart rate variability and QT interval can vary at different times of the day [9, 22–24]. Therefore, in this study, morning and evening ECG recordings were taken to assess the possible daily variability in autonomic regulation. Patients’ body temperatures were recorded during the ECG recording. Patients with fever on the first day of hospitalization were given antipyretic treatment. ECG recordings were taken in the morning and evening during fever-free periods.

#### Electrocardiographic recording

A calibrated digital ECG system was used to obtain a standard 12-lead surface ECG at a paper speed of 25 mm/s and an amplitude of 10 mm/mV. To assess circadian variation, ECG recordings were made at two standard time points: morning (08:00–10:00) and evening (18:00–20:00). These time intervals were selected to reflect physiologically distinct phases of autonomic circadian modulation. All recordings were obtained in a quiet room with controlled ambient temperature after at least 10 min of rest in the supine position. Electrocardiographic measurements were performed by a single investigator.

#### Electrocardiographic analysis

ECG measurements were performed manually using electronic measuring instruments with magnification on high-resolution digital ECG recordings. Heart rate, PR interval, QRS duration, QT interval, and corrected QT (QTc) were measured from a standard 12-lead ECG. To reduce measurement error, all measurements were evaluated by averaging three consecutive cardiac cycles. The QT interval was measured from the beginning of the QRS complex to the end of the T wave. The end of the T wave was defined as the point where the tangent drawn to the descending limb of the T wave intersects the isoelectric line (tangent method). This method was preferred to reduce measurement variability, especially in cases where the T wave termination was not clearly defined. The QT interval was corrected for heart rate using the Bazett formula (QTc) [25].

The Tp-e interval was defined as the time between the peak and the end of the T wave. Measurements were performed in precordial leads (V2–V6). For analysis, the derivation that allowed for the clearest evaluation of T wave morphology was selected. The peak of the T wave was considered to be the point where the wave reached its maximum amplitude. If necessary, the end of the T wave was determined using the tangent method. To minimize measurement variability, all measurements were performed manually using electronic measuring calipers on magnified high-resolution digital ECG recordings. Leads with unclear T wave termination, low amplitude, or significant basal artifacts were excluded from the analysis. Tp-e dispersion was calculated as the difference between the maximum and minimum Tp-e values obtained in the measurable derivations. Additionally, Tp-e/QT and Tp-e/QTc ratios were calculated to assess ventricular repolarization heterogeneity [26, 27].

#### Laboratory and clinical data

At the time of diagnosis in children with pneumonia and during evaluation in the healthy control group, routine laboratory parameters, including complete blood count and C-reactive protein (CRP) levels, were obtained**.** CRP was the only inflammatory marker evaluated in this study. To exclude potential electrolyte and metabolism-related effects on ventricular repolarization parameters, the baseline electrolyte levels of all participants were confirmed to be within normal reference ranges.

#### Use of artificial intelligence tools

Artificial intelligence-based tools (ChatGPT, OpenAI) were used solely for language editing and improvement of the manuscript. These tools were not used in the study design, data collection, data analysis, or interpretation of the results. The authors take full responsibility for the content of the manuscript.

#### Statistical analysis

Continuous variables were presented as mean ± standard deviation. Data distribution was assessed using the Kolmogorov–Smirnov test and visual methods (histograms and Q–Q plots). Non-parametric tests were used for variables that did not show a normal distribution. Age distribution between groups was compared using the Mann–Whitney *U* test, and gender distribution was compared using the chi-square test. Intra-group morning–evening comparisons were performed using the paired *t*-test, depending on the data distribution. The effect of time, the effect of group (CAP children versus healthy control group), and the time × group interaction in the morning and evening measurements were analyzed using a linear mixed model. The time variable was treated as a within-subject factor, and the group variable as between-subjects factor. The relationships between Tp–e and Tp–e dispersion and QTc were evaluated separately for morning and evening measurements using Spearman correlation analysis. Parameters that did not show variability over time were not included in the interaction analyses. Statistical analyses were performed using IBM SPSS Statistics software (IBM Corp., Armonk, NY, USA). Two-tailed *p* < 0.05 was considered statistically significant in all tests.

## Results

The median age of the CAP group was 5.0 (4.0–8.5) years, while the median age of the control group was 7.0 (5.0–9.0) years. There was no statistically significant difference between the two groups in terms of age distribution (*p* = 0.087). Similarly, there was no statistically significant difference in gender distribution (*p* = 0.935).

When comparing the morning and evening electrocardiographic measurements of the groups, no significant differences were found between morning and evening measurements for most parameters in the control group (all *p* > 0.05). However, in the control group, the frontal QRS–T angle was lower in the evening measurements compared to the morning measurements, and this difference was statistically significant (*p* = 0.049). In the pneumonia group, no significant changes were observed between morning and evening measurements in terms of heart rate, P wave duration, PR interval, QRS duration, QT, QTc, Tp-e interval, Tp-e dispersion, and Tp-e/QT and Tp-e/QTc ratios (all *p* > 0.05). However, evening measurements showed borderline statistical trends toward increases or decreases in heart rate (*p* = 0.098), P wave duration (*p* = 0.090), QTc (*p* = 0.083), and Tp-e interval (*p* = 0.108). Overall, the number of parameters showing a significant circadian variation between morning and evening measurements was limited in both groups, and electrocardiographic repolarization and conduction parameters were largely stable (Table 1).

**Table 1:** Morning and evening electrocardiographic parameters in healthy controls and children with community-acquired pneumonia

Parameters	Control AM (mean ± SD)	Control PM (mean ± SD)	*p*	Pneumonia AM (mean ± SD)	Pneumonia PM (mean ± SD)	*p*
Heart rate	97.0 ± 23.4	93.9 ± 22.4	0.194	127.0 ± 20.8	135.8 ± 26.0	0.098
P-wave duration	79.4 ± 3.2	79.8 ± 1.5	0.323	72.6 ± 16.6	68.1 ± 11.4	0.090
PR interval	134.1 ± 24.4	127.2 ± 14.7	0.062	109.6 ± 24.5	103.6 ± 17.6	0.213
QRS duration	83.3 ± 11.1	82.0 ± 9.4	0.475	69.9 ± 13.1	70.1 ± 5.7	0.913
QT interval	344.7 ± 36.8	347.7 ± 34.9	0.520	295.9 ± 29.3	290.3 ± 30.8	0.386
QTc	395.6 ± 19.6	393.6 ± 22.5	0.595	428.1 ± 22.2	417.7 ± 25.2	0.083
Tp-e	59.7 ± 6.2	58.0 ± 6.0	0.163	65.3 ± 5.3	63.1 ± 7.4	0.108
Tp–e dispersion	19.4 ± 2.4	20.1 ± 1.9	0.098	23.8 ± 3.7	23.5 ± 4.3	0.436
Frontal QRS-T	30.2 ± 18.2	24.9 ± 16.1	**0.049**	28.6 ± 29.8	22.2 ± 6.8	0.196
Tp-e/QT	0.18 ± 0.03	0.17 ± 0.03	0.137	0.22 ± 0.03	0.22 ± 0.04	0.778
Tp-e/QTc	0.15 ± 0.02	0.15 ± 0.02	0.668	0.15 ± 0.02	0.15 ± 0.02	0.688

Data are presented as mean ± standard deviation (SD). Morning and evening values were compared within each group. *p* values refer to the comparison between morning and evening measurements. QTc was calculated using Bazett’s formula. Tp–e represents the interval from the peak to the end of the T wave; Tp–e dispersion indicates the difference between the maximum and minimum Tp–e values across leads. *p* < 0.05 was considered statistically significant. AM, morning; PM, evening. *p* values refer to within-group comparisons between morning and evening measurements

The effects of time, group, and time × group interaction on electrocardiographic parameters are summarized in Table 2. The group effect was found to be significant for heart rate, P wave duration, PR interval, QRS duration, QT interval, QTc interval, Tp-e interval, Tp-e dispersion, and Tp-e/QT ratio (all *p* < 0.001). In contrast, the group effect was not significant for the Tp-e/QTc ratio and frontal QRS–T angle (*p* > 0.05). The time effect was significant for the PR interval (*p* = 0.030), Tp-e interval (*p* = 0.033), and frontal QRS–T angle (*p* = 0.027). No significant changes were detected between morning and evening measurements for other electrocardiographic parameters. The time × group interaction was significant only for heart rate (*p* = 0.026) and P wave duration (*p* = 0.036). No time × group interaction was observed for other parameters. No ST segment depression or T wave inversion was observed in any participant throughout the study.

**Table 2:** Effects of time, group, and time × group interaction on electrocardiographic parameters

Parameters	Time effect (p)	Group effect (p)	Time × Group interaction (p)
Heart rate	0.288	** < 0.001**	**0.026**
P-wave duration	0.081	** < 0.001**	**0.036**
PR interval	0.030	** < 0.001**	0.876
QRS duration	0.705	** < 0.001**	0.583
QT interval	0.738	** < 0.001**	0.267
QTc	0.063	** < 0.001**	0.199
Tp-e	**0.033**	** < 0.001**	0.785
Tp–e dispersion	0.976	** < 0.001**	0.124
Tp-e/QT	0.250	** < 0.001**	0.642
Tp-e/QTc	0.391	0.353	0.715
Frontal QRS-T	**0.027**	0.528	0.823

Analyses were performed using linear mixed-effects models. Time (morning vs. evening) was treated as a within-subject factor, and group (healthy controls vs. pneumonia) as a between-subject factor. *p* values represent fixed effects for time, group, and time × group interaction. A *p* value < 0.05 was considered statistically significant

The relationships between Tp-e interval, Tp-e dispersion, and ratio-based repolarization indices (Tp-e/QT and Tp-e/QTc) and QTc were evaluated separately for morning and evening measurements (Table 3). The Tp-e interval and Tp-e dispersion did not show a significant correlation with QTc in morning and evening measurements (all *p* > 0.05). In contrast, the Tp-e/QT ratio showed a moderate and statistically significant correlation with QTc in morning measurements (*r* =  − 0.360, *p* = 0.031), while this relationship was not significant in evening measurements. The Tp-e/QTc ratio showed a strong and significant negative correlation with QTc in both morning and evening measurements (morning: *r* =  − 0.618, *p* < 0.001; evening: *r* =  − 0.427, *p* = 0.009).

**Table 3:** Correlations between QTc and Tp–e-related indices in the pneumonia group

Parameters	Time	Spearman *r*	*p*
**Tp–e interval**	Morning	− 0.130	0.451
	Evening	− 0.007	0.966
**Tp–e dispersion**	Morning	0.147	0.392
	Evening	0.016	0.925
**Tp–e/QT**	Morning	− 0.360	**0.031**
	Evening	0.263	0.122
**Tp–e/QTc**	Morning	− 0.618	** < 0.001**
	Evening	− 0.427	0.009

Spearman correlation analysis was performed to evaluate the associations between QTc and Tp–e-related repolarization indices, including Tp–e interval, Tp–e dispersion, Tp–e/QT, and Tp–e/QTc, in the pneumonia group. Morning and evening measurements were analyzed separately. Correlation coefficients are presented as Spearman’s r. A *p* value < 0.05 was considered statistically significant

No significant differences in serum electrolyte levels were found between children with CAP and healthy controls (Table 4). Although CRP levels tended to be higher in the pneumonia group, this difference was not statistically significant (*p* = 0.187): Serum sodium (*p* = 0.164), potassium (*p* = 0.334), chloride (*p* = 0.101), calcium (*p* = 0.858), phosphorus (*p* = 0.461), magnesium (*p* = 0.348), and glucose (*p* = 0.567) levels.

**Table 4:** Comparison of CRP and serum electrolyte levels between healthy children and children with community-acquired pneumonia

Parametre	Healthy controls	Children with community acquired pneumonia	*P*
CRP	6.08 ± 16.32	11.0 ± 17.20	0.187
Sodium	137.83 ± 2.37	137.08 ± 2.44	0.164
Potassium	4.35 ± 0.33	4.43 ± 0.48	0.334
Chloride	102.87 ± 1.88	102.09 ± 2.44	0.101
Calcium	9.92 ± 0.50	9.94 ± 0.87	0.858
Phosphorus	4.01 ± 0.44	4.08 ± 0.38	0.461
Magnesium	1.99 ± 0.27	1.93 ± 0.27	0.348
Glucose	93.15 ± 12.48	91.08 ± 20.13	0.567

Values are presented as mean ± SD.Comparisons between groups were performed using the independent samples t-test.A *p* value < 0.05 was considered statistically significant

## Discussion

Community-acquired pneumonia in childhood is associated with systemic inflammation and autonomic activation; however, its temporal effects on cardiac electrophysiology remain incompletely defined. In this study, CAP was associated with measurable changes in atrial conduction and ventricular repolarization during morning and evening assessments. While healthy children showed stable circadian patterns, CAP was characterized by limited but specific alterations in circadian modulation.

In pediatric ECG, the QRS axis shows age-dependent variation: newborns and infants generally have a more rightward axis, and this axis gradually shifts to the left during childhood, usually falling to adult-like ranges in later childhood [28]. In our study, the age distribution was similar among the groups, which minimizes potential age-related effects on the QRS axis and other electrophysiological measurements.

In healthy children, the consistency of electrocardiographic parameters between morning and evening measurements supports the maintenance of cardiac electrical stability under physiological conditions [8]. Circadian regulation of cardiac electrophysiology occurs through autonomic and intrinsic mechanisms [29]. In our study, circadian regulation was preserved in healthy children, but in children with CAP, the heart rate showed an increase rather than the expected daily decrease at both time points, suggesting impaired chronotropic control [30].

Electrocardiographic parameters are dynamically regulated by autonomic mechanisms and exhibit diurnal variation, shortening during daytime sympathetic predominance and lengthening at night [31, 32]. In our study, P-wave duration, PR interval, and QRS duration were shorter in children with CAP at both time points, suggesting a stable shift in atrial conduction rather than a time-dependent variation. This may be due to increased sympathetic activity or functional adaptations in atrial conduction during acute infection [8, 33, 34]. Although prolongation of conduction times has been reported in inflammatory processes, there is also evidence that acute autonomic changes may shorten atrial depolarization time [8, 33, 34].

Age may influence the electrical QRS axis, with younger children tending to have a more rightward axis. This was considered when interpreting the electrocardiographic findings in our study.

The time × group interaction detected for heart rate and P wave duration indicates that the physiological evening slowdown is markedly weakened in children with CAP. The absence of this interaction in QRS duration and repolarization parameters suggests that the effect of pneumonia on circadian regulation is particularly evident through atrial and chronotropic responses. This finding suggests that the disease reshapes the normal diurnal pattern by suppressing it rather than completely eliminating the circadian rhythm. It is known that changes in autonomic nervous system activity can affect atrial conduction and sinus node activity by modulating ion channel expression and function in atrial myocytes [8, 35–37].

Ventricular repolarization is a key component of circadian cardiac electrophysiology, regulated by the interaction between autonomic activity and intrinsic cardiac clock mechanisms [6–8]. Circadian clock genes contribute to the temporal stability of repolarization through the modulation of cardiac ion channels [9]. In our study, ventricular repolarization parameters showed limited daily variation in healthy children but differed between groups; this suggests that the observed changes are primarily disease-related rather than time-dependent [38].

In our study, the Tp-e/QT and Tp-e/QTc ratios were evaluated as normalized indicators of ventricular repolarization heterogeneity relative to the QT interval. The similar course of these ratios between morning and evening measurements in healthy children suggests that repolarization heterogeneity is maintained in a temporally stable manner under physiological conditions. The fact that these ratios were higher in children with pneumonia compared to the control group, but did not show a significant change depending on the time of measurement, suggests that the increase in repolarization heterogeneity persists without forming a diurnal pattern. An important and novel observation of this study is that it addresses the time-dependent variation in the relationships between repolarization parameters rather than their absolute values. Notably, in children with CAP, significant correlations between QTc and Tp-e-based ratios were present in the morning but weakened or disappeared in the evening. This suggests that circadian modulation may have a greater effect on the intrinsic consistency of repolarization dynamics than on individual ECG measurements. Clinically, these findings highlight the importance of considering measurement timing when interpreting repolarization markers, particularly in acute inflammatory conditions.

The frontal QRS-T angle provides an integrated measure of spatial ventricular depolarization-repolarization heterogeneity and has been associated with adverse outcomes in various cardiac conditions [38]. In this study, a small but statistically significant morning-evening difference in the frontal QRS-T angle was observed in healthy children, suggesting preserved physiological modulation of ventricular electrical alignment. In contrast, no significant temporal variation was observed in children with CAP. Importantly, absolute QRS-T angle values remained below established pathological thresholds in both groups, suggesting that the observed changes are unlikely to reflect structural myocardial damage. Instead, these findings support the notion that electrophysiological alterations associated with CAP may be driven by functional, reversible, and primarily transient autonomic and inflammatory effects [39–41].

All participants included in the study had electrolyte levels within the normal range and comparable between groups. Similarly, no significant difference was observed in CRP levels. This finding may reflect the limitations of using a single inflammatory biomarker, as CRP is a relatively late-phase reactant and may not adequately reflect acute inflammatory or neuroimmune processes. The relatively modest CRP elevation may be related to the inclusion of clinically stable, non-severe CAP cases. Therefore, the observed ECG changes may not correlate perfectly with CRP levels and may be related to autonomic mechanisms.

Pathophysiologically, systemic inflammation, sympathetic overactivity, and oxidative stress have been shown to affect both central circadian regulation and peripheral cardiac clock-gene expression [42–44]. It has been reported that inflammatory cytokines may disrupt molecular clock function, leading to changes in ion channel expression and impaired temporal coordination of cardiac electrical activity [45]. Although clock gene expression or autonomic biomarkers were not directly assessed in this study, the observed electrophysiological changes are consistent with partial circadian modulation rather than complete circadian collapse. This distinction is particularly important in pediatric populations. Clinically, our findings demonstrate that cardiac electrophysiological assessment in children with CAP should not rely solely on single parameters such as QT or QTc. A multidimensional approach including atrial conduction indices, repolarization heterogeneity markers, and ratio-based parameters provides a more comprehensive assessment of cardiac electrical adaptation during acute infection. Measurable changes in heart rate regulation, atrial conduction, and ventricular repolarization have been observed even in clinically stable children, indicating subclinical cardiac involvement that may be overlooked in routine assessment [46].

In particular, increases in Tp-e and Tp-e-based ratios suggest a transient increase in ventricular repolarization heterogeneity during acute infection, even in the absence of significant electrolyte imbalance or structural heart disease. The selective attenuation of diurnal modulation, while overall circadian regulation is largely preserved, suggests that single-time-point ECG assessments may underestimate time-dependent electrophysiological changes. In conclusion, in children hospitalized with pneumonia, particularly those with tachycardia, borderline QT values, or unexplained arrhythmias, careful evaluation of ECG findings, considering temporal variability and autonomic tone, is necessary.

In conclusion, measurable changes in atrial conduction and ventricular repolarization are observed in children with CAP compared to healthy controls. Although global circadian regulation is largely preserved, selective changes in chronotropic and atrial conduction patterns suggest a reshaping rather than a loss of physiological rhythm. Time-dependent variability in QTc and Tp-e-based indices demonstrates the importance of temporal context in ECG interpretation during acute pediatric infections.

## Study limitations

This study has several limitations. First, ECG recordings were obtained at only two time points instead of continuous monitoring. Second, the sample size was relatively small. Third, the etiological distinction between viral and bacterial pneumonia was primarily based on clinical assessment. Fourth, due to the cross-sectional design, the normalization of cardiac parameters during the recovery period could not be evaluated. Furthermore, since patients were not divided into subgroups of mild and moderate pneumonia, potential differences in ECG parameters between subgroups could not be investigated. The relationship between changes in the electrical axis in the frontal plane and pulmonary air trapping or changes in lung mechanics in patients with pneumonia was not evaluated. Air trapping, which can be seen especially in viral pneumonias, is a potential factor that may influence changes in the cardiac electrical axis; however, since pulmonary mechanical effects or air trapping were not evaluated by imaging in our study, it was not possible to establish this relationship. Future studies involving continuous ECG monitoring and circadian biomarkers may provide deeper insights into the temporal dynamics of cardiac electrophysiology during acute infections.

## Data Availability

Data are available from the corresponding author upon reasonable request.

## Abbreviations

CAP: Community-acquired pneumonia
CRP: C-reactive protein
ECG: Electrocardiography
HR: Heart rate
PR: PR interval
QTc: Corrected QT interval
QRS: QRS duration
Tp-e: T peak–T end interval
